# Describing and Quantifying Asthma Comorbidty: A Population Study

**DOI:** 10.1371/journal.pone.0034967

**Published:** 2012-05-07

**Authors:** Andrea S. Gershon, Jun Guan, Chengning Wang, J. Charles Victor, Teresa To

**Affiliations:** 1 Institute for Clinical Evaluative Sciences, Toronto, Ontario, Canada; 2 The Hospital For Sick Children, Toronto, Ontario, Canada; 3 University of Toronto, Toronto, Ontario, Canada; The University of Edinburgh, United Kingdom

## Abstract

**Background:**

Asthma comorbidity has been correlated with poor asthma control, increased health services use, and decreased quality of life. Managing it improves these outcomes. Little is known about the amount of different types of comorbidity associated with asthma and how they vary by age.

**Methodology/Principal Findings:**

The authors conducted a population study using health administrative data on all individuals living in Ontario, Canada (population 12 million). Types of asthma comorbidity were quantified by comparing physician health care claims between individuals with and without asthma in each of 14 major disease categories; results were adjusted for demographic factors and other comorbidity and stratified by age. Compared to those without asthma, individuals with asthma had higher rates of comorbidity in most major disease categories. Most notably, they had about fifty percent or more physician health care claims for respiratory disease (other than asthma) in all age groups; psychiatric disorders in individuals age four and under and age 18 to 44; perinatal disorders in individuals 17 years and under, and metabolic and immunity, and hematologic disorders in children four years and under.

**Conclusion/Significance:**

Asthma appears to be associated with significant rates of various types of comorbidity that vary according to age. These results can be used to develop strategies to recognize and address asthma comorbidity to improve the overall health of individuals with asthma.

## Introduction

Asthma has been associated with various types of comorbidity, from coronary artery to psychiatric disease, that place significant burden on patients [1]–[3]. Asthma comorbidity has been correlated with poor asthma control, increased health care use, and decreased quality of life, and managing it has been shown to significantly improve these outcomes [4]–[9]. Despite this, there is still little known and relatively little attention paid to diagnosing and treating asthma comorbidity [10]. This is in contrast to other chronic disease comorbidity–such as renal disease among individuals with diabetes and hypercholesterolemia among individuals with coronary artery disease–that have been well studied and are routinely identified and treated accordingly.

Knowledge of the full spectrum of diseases that accompany asthma, the diseases that have the greatest impact and, as asthma can last a lifetime, how they vary by age would help physicians, other health care providers, and policy makers develop strategies to recognize, prioritize, and manage asthma comorbidity effectively. To the best of our knowledge, no previous studies have systematically investigated and measured the impact of different types of comorbidity on individuals with asthma in different life stages. Therefore, we conducted the current study to characterize and quantify different types of asthma comorbidity in individuals of different ages.

## Methods

### Ethics Statement

The study was approved by the institutional review boards at Sunnybrook Health Sciences Centre and The Hospital for Sick Children, Toronto, Ontario. For the purposes of this research informed consent was not required. The Institute for Clinical Evaluative Sciences (ICES) is named as a prescribed entity in Section 45 of the *Personal Health Information Protection Act* (PHIPA – Regulation 329/04, Section 18). Under this designation, ICES can receive and use health information without consent for purposes of analysis and compiling statistical information about the Ontario health care system.

**Table 1:** Characteristics of individuals with and without asthma living in Ontario, Canada.

Characteristic	Individuals with asthma	Individuals without asthma	*P*-value for equivalence*
N	1,477,575	10,143,134	
Age, years (mean (standard deviation))	31.8 (22.8)	38.2 (21.6)	1.000
Female (%)	53.2	50.6	0.985
Socioeconomic Status			
- Quintile 1 (Lowest)	19.7	18.9	<0.001
- Quintile 2	19.9	19.7	
- Quintile 3	20.2	20.1	
- Quintile 4	20.4	20.5	
- Quintile 5 (Highest)	19.5	20.4	
Rural (versus urban) residence (%)	11.8	13.2	<0.001
COPD (%)	16.0	4.7	1.000

COPD, Chronic Obstructive Pulmonary Disease.

* Equivalence tests performed. Significant *P*-values indicate that distributions are *‘practically equivalent’* to within +/− two percentage points for proportions and 0.75 years for age.

### Study Design and Setting

We conducted a retrospective population study using universal, health administrative data from Ontario, Canada–the largest province of Canada with a multicultural population of approximately 12 million [11].

### Data Sources

Residents of Ontario have universal public health insurance under the Ontario Health Insurance Plan, the single payer for all medically necessary services across the full spectrum of residents, providers and hospitals. Service details are captured in health administrative databases which can be linked on an individual level to provide a complete health services profile for each resident. Three Ontario population-based health administrative databases were used to identify individuals with asthma and measure their comorbidity through their health services use. The Ontario Health Insurance Plan physician services claims database contains information about all services provided by fee-for-service physicians and “shadow-billings” for physicians paid under alternate payment plans. Each physician claim is accompanied by one International Classification of Disease 9^th^ Revision (ICD-9) diagnostic code. The Canadian Institute for Health Information Discharge Abstract Database contains information on all hospitalizations in the province. Finally, the Ontario Registered Persons Database maintains demographic information on all individuals living in Ontario.

**Figure 1:**
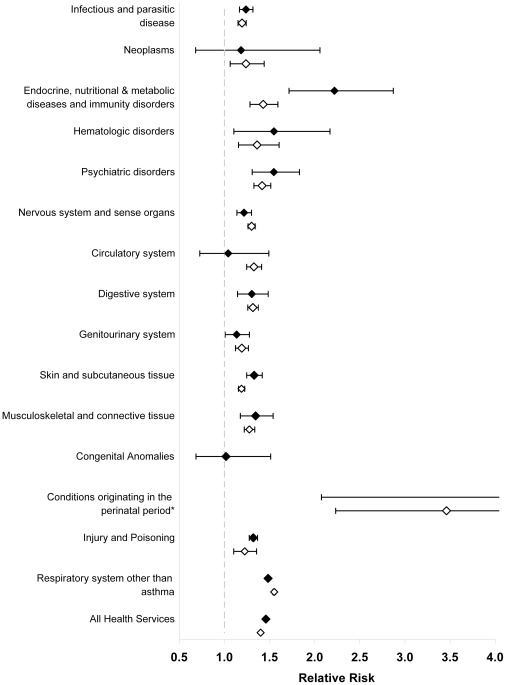
Adjusted relative risks and 95% confidence intervals of comorbidity, as indicated by health services use, among individuals four years and younger and five to 17 years with compared to without asthma in 14 disease categories. Solid diamonds represent relative risks for individuals four years and younger; hollow diamonds represent relative risks for individuals five to 17 years. All analyses were adjusted for age, sex, socioeconomic status, rural/urban place of residence, a co-diagnosis of COPD, and other comorbidity as indicated by ICD-9 category. * Relative risk for conditions originating in the perinatal period among individuals zero to four years old: 5.03, 95% CI: 2.08 to 12.21 and among individuals five to 17 years old: 3.46 (2.23 to 5.36).

**Table 2:** Rates of health services claims among individuals with and without asthma in 14 disease categories, according to age (Pediatric population).

	Health Services Claims per 1000 person years						
	4 years and younger			5 to 17 years			
	Individuals with asthma	Individuals without asthma	Absolute rate difference		Individuals with asthma	Individuals without asthma	Absolute rate difference
Disease Category	(N = 90,387)	(N = 536,432)	(95% confidence interval)		(N = 464,068)	(N = 1,555,638)	(95% confidence interval)
Infectious and parasitic disease	423.0	330.6	92.4 (90.6, 94.2)		256.4	208.7	47.7 (47.1, 48.4)
Neoplasms	38.3	33.7	4.7 (4.1, 5.2)		43.5	39.5	4.1 (3.8, 4.3)
Endocrine, nutritional, and metabolic diseases, and immunity disorders	47.4	30.8	16.6 (16.0, 17.2)		76.9	56.7	20.2 (19.8, 20.6)
Hematologic disorders	32.0	22.7	9.3 (8.8, 9.8)		25.6	21.8	3.8 (3.6, 4.0)
Psychiatric disorders	222.1	133.5	88.6 (87.3, 89.9)		431.5	316.3	115.2 (114.3, 116.0)
Nervous system and sense organs	744.0	593.8	150.2 (147.7, 152.6)		333.8	254.9	78.9 (78.1, 79.6)
Circulatory system	46.4	29.9	16.6 (15.9, 17.2)		71.0	54.9	16.2 (15.8, 16.5)
Digestive system	228.2	172.2	56.0 (54.6, 57.3)		207.9	160.4	47.5 (46.9, 48.0)
Genitourinary system	116.9	101.1	15.8 (14.8, 16.8)		161.4	152.3	9.1 (8.5, 9.6)
Skin and subcutaneous tissue	337.1	260.9	76.2 (74.6, 77.9)		371.1	300.3	70.8 (70.0, 71.6)
Musculoskeletal system and connective tissue	74.6	54.3	20.3 (19.6, 21.1)		178.9	138.4	40.4 (39.9, 41.0)
Congenital anomalies	29.7	20.3	9.5 (9.0, 9.9)		16.2	10.9	5.3 (5.1, 5.5)
Conditions originating in the perinatal period	7.9	3.0	4.9 (4.7, 5.2)		2.3	1.1	1.2 (1.1, 1.2)
Injury and poisoning	309.8	236.0	73.8 (72.3, 75.4)		444.1	344.3	99.8 (98.9, 100.7)
Respiratory system other than asthma	1571.7	1058.1	513.6 (510.1, 517.1)		817.6	520.5	297.1 (296.0, 298.3)

**Table 3:** Rates of health services claims among individuals with and without asthma in 14 disease categories, according to age (Adult population).

	Health Services Claims per 1000 person years								
	18 to 44 years			45 to 64 years			65 years and older		
	Individuals with asthma	Individuals without asthma	Absolute rate difference	Individuals with asthma	Individuals without asthma	Absolute rate difference	Individuals with asthma	Individuals without asthma	Absolute rate difference
Disease Category	(N = 492,044)	(N = 4,154,792)	(95% confidence interval)	(N = 264,150)	(N = 2,583,341)	(95% confidence interval)	(N = 166,926)	(N = 1,312,931)	(95% confidence interval)
Infectious and parasitic disease	239.1	171.9	67.3 (66.7, 67.8)	220.2	137.3	82.9 (82.2, 83.7)	290.7	199.1	91.7 (90.5, 92.8)
Neoplasms	148.1	128.0	20.1 (19.7, 20.6)	535.4	458.5	76.9 (75.7, 78.1)	1111.6	1027.5	84.0 (81.7, 86.4)
Endocrine, nutritional, and metabolic diseases, and immunity disorders	329.0	243.2	85.9 (85.2, 86.6)	930.0	667.7	262.3 (260.7, 263.8)	1093.0	966.1	126.9 (124.6, 129.2)
Hematologic disorders	57.2	42.5	14.7 (14.4, 15.0)	114.2	72.0	42.2 (41.7, 42.8)	317.8	246.2	71.6 (70.3, 72.8)
Psychiatric disorders	1273.2	723.2	550.0 (548.7, 551.3)	1428.7	783.1	645.6 (643.6, 647.5)	1408.0	1257.0	150.9 (148.3, 153.6)
Nervous system and sense organs	443.4	300.7	142.7 (141.9, 143.5)	820.1	564.2	255.8 (254.4, 257.3)	1684.8	1426.1	258.7 (255.8, 261.6)
Circulatory system	382.9	281.5	101.4 (100.7, 102.2)	1661.3	1199.3	462.0 (459.9, 464.1)	4342.6	3573.0	769.6 (765.0, 774.3)
Digestive system	509.7	324.9	184.8 (183.9, 185.7)	829.5	529.6	299.9 (298.4, 301.3)	1236.9	882.3	354.6 (352.1, 357.1)
Genitourinary system	627.5	458.7	168.8 (167.8, 169.7)	734.6	509.4	225.3 (223.9, 226.7)	1099.8	885.1	214.7 (212.4, 217.0)
Pregnancy, childbirth and the puerperium	310.2	241.6	68.6 (68.0, 69.3)	2.1	1.4	0.7 (0.6, 0.7)	na	na	na
Skin and subcutaneous tissue	365.3	270.8	94.5 (93.7, 95.2)	443.8	302.0	141.8 (140.7, 142.8)	598.4	479.3	119.1 (117.3, 120.8)
Musculoskeletal system and connective tissue	618.4	398.1	220.3 (219.3, 221.2)	1383.6	807.8	575.8 (573.9, 577.7)	1732.3	1267.2	465.1 (462.2, 468.0)
Injury and poisoning	510.5	342.1	168.3 (167.5, 169.2)	653.6	413.2	240.4 (239.1, 241.7)	945.9	720.2	225.7 (223.6, 227.9)
Respiratory system other than asthma	979.4	497.6	481.8 (480.6, 483.0)	1588.9	536.6	1052.3 (1050.3, 1054.3)	2853.5	926.9	1926.7 (1923.0, 1930.3)

na, not applicable.

### Study Population

All individuals living in Ontario on April 1, 2003 (the index date) were followed until March 31, 2008. Those who died or left the province were excluded. Individuals with asthma were identified using a previously validated asthma case definition based on health administrative data [12]. The case-definition of two or more asthma physician visits within two consecutive years and/or one or more asthma hospitalization yielded 89% sensitivity and 72% specificity in children (aged less than17 years) and 84% sensitivity and 76% specificity in adults (aged 18 years or over) when compared to clinical evaluation. More details of the asthma case definition and examples of its use in other studies can be found elsewhere [12]–[14]. Subjects were stratified by age into the following groups: less than four years (preschool), five to 17 years (children and adolescence), 18 to 44 years (younger adults), 45 to 64 years (middle aged adults) and 65 years and older (older adults).

**Figure 2:**
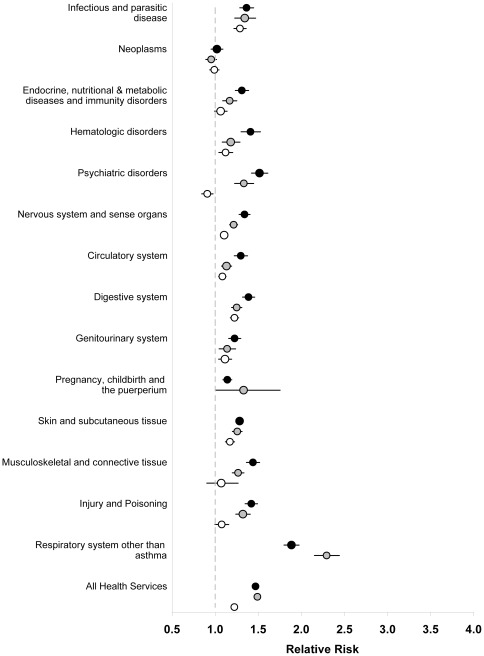
Adjusted relative risks and 95% confidence intervals of comorbidity, as indicated by health services use, among individuals age 18 to 44 years, 45 to 64 years and 65 years and older with compared to without asthma in 14 disease categories. Solid black circles represent relative risks for individuals 18 to 44 years; solid grey circles represent relative risks for individuals 45 to 64 years; hollow circles represent relative risks for individuals age 65 years and older. All analyses were adjusted for age, sex, socioeconomic status, rural/urban place of residence, a co-diagnosis of COPD, and other comorbidity as indicated by ICD-9 category.

### Outcome Variables

The primary outcome was amount of comorbidity in individuals with asthma which was compared to amount of comorbidity in those without asthma. As a direct correlation between amount of comorbidity and health service claims has been previously well validated, amount of comorbidity was examined through physician claims [15], [16]. Physician claims capture health service use in the ambulatory, emergency department and hospitalization setting. Comorbidity was stratified by diagnosis according to 14 ICD-9 disease categories [17]. ICD-9 categories that were vague and/or heterogeneous (such as symptoms, signs and ill-defined conditions and supplementary classification) were not examined. In secondary analysis, a number of specific conditions previously shown to be associated with asthma were also examined [1]. Details of the 14 ICD-9 disease categories and specific conditions, their associated ICD-9 codes, and the populations in which they were studied are presented in Supplemental Table S1.

### Potential Confounding Variables

A number of demographic and clinical variables obtained from the health administrative data were adjusted for in multivariable analysis (Table 1). Socioeconomic status was inferred from neighborhood income derived from postal codes and census data [7], [18]. Rural status was based on Statistics Canada's definition of rurality [19]. Because it could potentially be misclassified as asthma, a co-diagnosis of chronic obstructive pulmonary disease (COPD) was also derived from health administrative data and controlled for [20]. Adjustment for other comorbidity as indicated by ICD-9 category was also done.

### Statistical Analyses

Frequencies and proportions were used to report socio-demographic characteristics. Normal approximation (z) tests for equivalence were conducted to determine if groups were statistically equivalent, using a difference margin of two percent absolute difference (i.e. significant p-values (p<0.05) indicated that the true difference in proportions was less than +/− 2%). For each ICD-9 disease category and condition studied absolute counts of physician claims and crude physician claim rates (per 1000 person years) were calculated for individuals with and without asthma. Absolute differences and their gamma-based 95% confidence intervals were also calculated. Adjusted relative risks of comorbidity in individuals with compared to those without asthma (and their 95% confidence intervals) were calculated using multivariable Poisson regression adjusting for age, sex, socioeconomic status, rural/urban place of residence, a co-diagnosis of COPD, and other comorbidity as indicated by ICD-9 categories. An adjusted relative risk more than or equal to one indicated that physician claims for a comorbidity were more common in individuals with compared to those without asthma and a relative risk less than one indicated the opposite. All analyses were stratified by age group and conducted with SAS version 9.2 for UNIX systems (Cary, North Carolina).

### Sensitivity Analysis

To assess whether it was plausible that misclassification of asthma due to less than perfect specificity of the case definition was responsible for the observed results, an array approach was used [21]. The inclusion of misclassified people could have potentially led to incorrect results if the misclassified people were healthier (or less healthy) than those correctly identified. Therefore, to determine if this influenced the results, we estimated the true relative risks when different assumptions about the health of those who were misclassified relative to those who truly had asthma were made. We were specifically Interested in what assumptions caused the relative risks to be reduced to 1.0 (or no increased risk).

**Figure 3:**
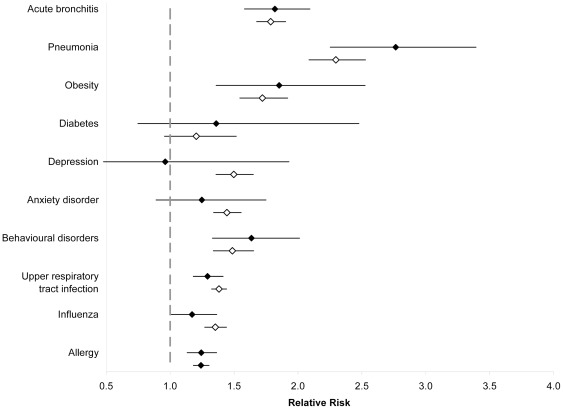
Adjusted relative risks and 95% confidence intervals of comorbidity, as indicated by health services use, use among individuals 4 years and younger and 5 to 17 years with compared to without asthma for specific disease conditions. Solid diamonds represent relative risks for individuals four years and younger; hollow diamonds represent relative risks for individuals five to 17 years. All analyses were adjusted for age, sex, socioeconomic status, rural/urban place of residence, a co-diagnosis of COPD, and other comorbidity as indicated by ICD-9 category.

**Table 4:** Rates of health services claims among individuals with and without asthma for specific disease conditions, according to age (Pediatric population).

	Health Services Claims per 1000 person years					
	4 years and younger			5 to 17 years of age		
	Individuals with asthma	Individuals without asthma	Absolute rate difference	Individuals with asthma	Individuals without asthma	Absolute rate difference
Specific Condition			(95% confidence interval)			(95% confidence interval)
Pneumonia	77.4	32.1	45.3 (44.5, 46.0)	22.9	10.6	12.3 (12.1, 12.5)
Influenza	32.6	24.5	8.1 (7.6, 8.6)	20.9	15.0	5.9 (5.8, 6.1)
Upper Respiratory Tract Infection	787.9	609.1	178.8 (176.3, 181.3)	362.5	259.3	103.2 (102.4, 104.0)
Depression	1.7	1.0	0.6 (0.5, 0.7)	30.1	21.9	8.2 (8.0, 8.4)
Anxiety disorder	40.9	26.9	13.9 (13.4, 14.5)	178.8	136.1	42.7 (42.2, 43.3)
Behavioural disorders	77.0	43.4	33.6 (32.8, 34.3)	65.0	38.9	26.1 (25.7, 26.4)
Obesity	11.7	6.2	5.5 (5.2, 5.8)	20.1	12.7	7.4 (7.2, 7.6)
Allergy	183.1	144.1	39.0 (37.8, 40.2)	120.4	91.1	29.3 (28.8, 29.7)
Acute bronchitis	179.9	101.5	78.4 (77.3, 79.6)	92.4	49.6	42.8 (42.4, 43.1)
Diabetes	7.3	5.6	1.7 (1.5, 2.0)	18.1	14.8	3.3 (3.1, 3.5)

**Table 5:** Rates of health services claims among individuals with and without asthma for specific disease conditions, according to age (Adult population).

	Health Services Claims per 1000 person years								
	18 to 44 years			45 to 64 years			65 years and older		
	Individuals with asthma	Individuals without asthma	Absolute rate difference	Individuals with asthma	Individuals without asthma	Absolute rate difference	Individuals with asthma	Individuals without asthma	Absolute rate difference
Specific Condition			(95% confidence interval)			(95% confidence interval)			(95% confidence interval)
Angina	9.5	6.6	3.0 (2.9, 3.1)	91.8	64.2	27.5 (27.0, 28.0)	273.6	211.2	62.4 (61.3, 63.6)
Cataracts	4.6	3.7	0.9 (0.8, 1.0)	96.2	66.6	29.6 (29.1, 30.1)	449.1	394.7	54.4 (52.9, 55.9)
Osteroporosis	4.6	3.2	1.4 (1.3, 1.4)	44.6	36.6	8.0 (7.7, 8.4)	93.0	83.0	10.0 (9.3, 10.7)
Glaucoma	8.4	7.4	1.1 (1.0, 1.2)	70.8	54.5	16.3 (15.9, 16.8)	204.4	179.4	25.0 (24.0, 26.0)
Myocardial infarction	15.5	13.1	2.5 (2.3, 2.6)	154.3	119.9	34.4 (33.8, 35.1)	549.9	456.2	93.7 (92.0, 95.3)
Pneumonia	36.2	14.7	21.5 (21.3, 21.7)	107.1	34.6	72.6 (72.0, 73.1)	435.2	182.4	252.8 (251.3, 254.2)
Influenza	21.4	14.7	6.8 (6.6, 6.9)	36.5	23.6	12.9 (12.6, 13.2)	54.9	47.9	7.0 (6.5, 7.5)
Upper Respiratory Tract Infection	303.3	196.8	106.5 (105.8, 107.1)	274.5	146.7	127.8 (127.0, 128.7)	254.4	148.0	106.4 (105.3, 107.5)
Depression	131.1	68.4	62.7 (62.2, 63.1)	154.5	79.4	75.1 (74.4, 75.7)	94.3	66.9	27.5 (26.8, 28.1)
Anxiety disorder	678.7	392.7	286.0 (285.0, 287.0)	819.0	446.3	372.7 (371.2, 374.2)	491.7	347.9	143.8 (142.2, 145.4)
Lung Cancer	59.5	34.2	25.3 (25.0, 25.6)	73.8	36.6	37.2 (36.8, 37.7)	21.2	13.5	7.7 (7.4, 8.0)
Obesity	3.1	2.3	0.8 (0.8, 0.9)	54.1	33.6	20.6 (20.2, 20.9)	147.8	91.7	56.1 (55.2, 56.9)
Allergy	112.6	81.8	30.7 (30.3, 31.1)	126.0	84.3	41.8 (41.2, 42.3)	156.6	119.3	37.4 (36.5, 38.3)
Acute bronchitis	3.1	2.3	0.8 (0.8, 0.9)	54.1	33.6	20.6 (20.2, 20.9)	147.8	91.7	56.1 (55.2, 56.9)
Diabetes	112.6	81.8	30.7 (30.3, 31.1)	126.0	84.3	41.8 (41.2, 42.3)	156.6	119.3	37.4 (36.5, 38.3)

## Results

### Study Population

There were 11,903,512 individuals living in Ontario in 2003 who were followed for five years. After excluding 282,803 who met at least one exclusion criterion (i.e. developed asthma, left the province, or died in the follow-up period) a study population of 1,477,575 (12.7%) individuals with and 10,143,134 (86.3%) individuals without asthma remained.

On average, individuals with asthma were younger (32 years compared to 38 years), more likely to be female, and more likely to have a co-diagnosis of COPD than those without asthma (Table 1). However, when examined by age group, compared to those without asthma, younger individuals (ages zero to four and five to 17 years) with asthma were more likely to be male and live in an urban region; individuals in the three older age groups (18 to 44, 45 to 64 and 65 years and older) with asthma were more likely to be female and have a co-diagnosis of COPD; and individuals in the two oldest age groups with asthma were more likely to live in a lower income neighborhood (data not shown).

**Figure 4:**
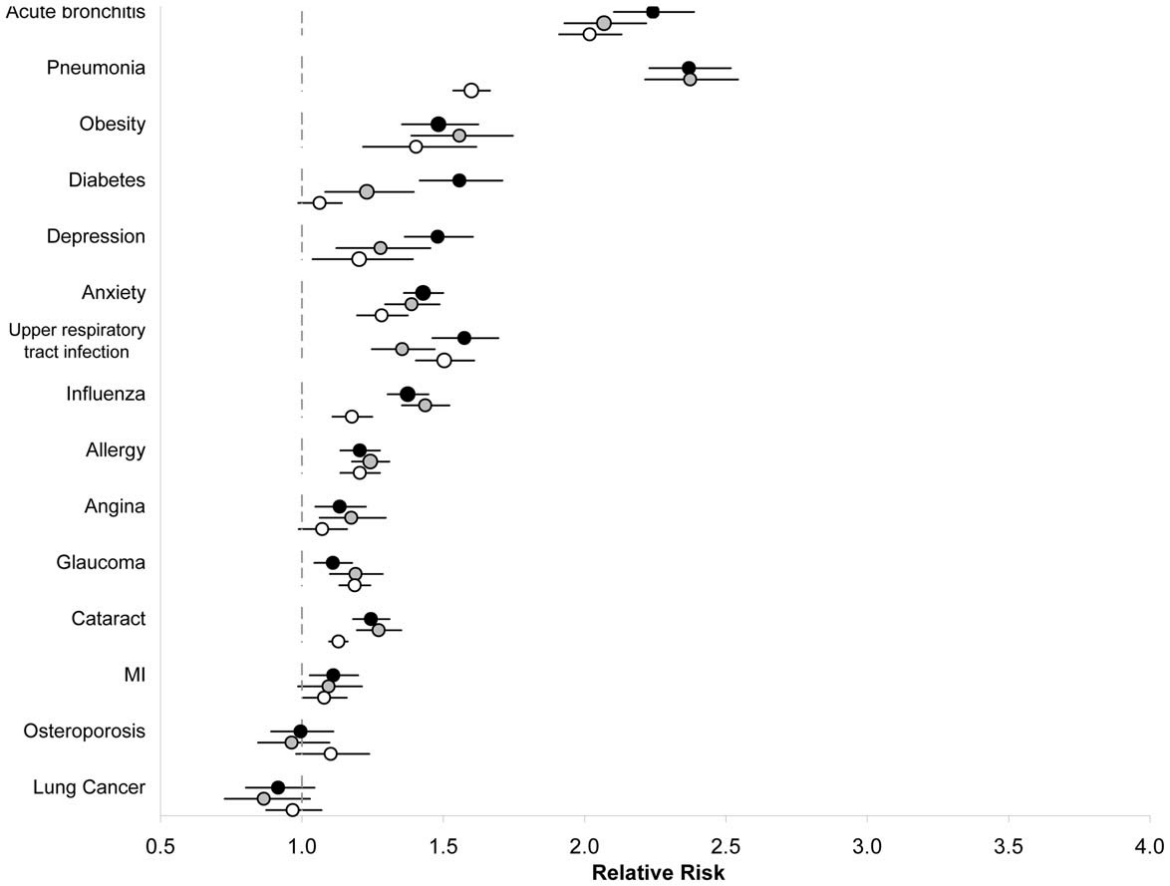
Adjusted relative risks and 95% confidence intervals of comorbidity, as indicated by health services use, among individuals age 18 to 44 years, 45 to 64 years and 65 years and older with compared to without asthma for specific disease conditions. Solid black circles represent relative risks for individuals 18 to 44 years; solid grey circles represent relative risks for individuals 45 to 64 years; hollow circles represent relative risks for individuals 65 years and older. All analyses were adjusted for age, sex, socioeconomic status, rural/urban place of residence, a co-diagnosis of COPD, and other comorbidity as indicated by ICD-9 category.

### Comorbidity in 14 Disease Categories

Compared to those without asthma, individuals with asthma had higher rates of comorbidity, as indicated by physician claims, in all 14 ICD-9 disease categories (Tables 2 and 3).

After adjusting for potential confounders, comorbidity in children four years and younger was 46% higher in those with compared to those without asthma (p<0.001; Figure 1). Higher comorbidity was found for most of the 14 disease categories but was highest (50% or greater) for respiratory disease (other than asthma); psychiatric disorders; metabolic and immunity disorders; hematologic disorders; and perinatal disorders (p<0.001 for each disease category).

Similarly, comorbidity was 40% higher among children aged five to 17 years with compared to without asthma (p<0.001; Figure 1). Significantly higher physician claim rates were found in all 14 disease categories (p<0.05) but were highest (more than 50% higher) for respiratory disease other than asthma and perinatal disorders.

**Table 6:** Rates of the 10 most common conditions in the Endocrine, nutritional, and metabolic diseases, and immunity disorders disease category in individuals with asthma age four years and younger and the corresponding rates in individuals without asthma.

	Health services claims per 1000 person years	
	Individuals with asthma	Individuals without asthma
Condition	(95% confidence interval)	(95% confidence interval)
Obesity	8.8 (8.6, 9.1)	4.6 (4.5, 4.7)
Diabetes mellitus	7.3 (7.1, 7.5)	5.6 (5.5, 5.6)
Other metabolic disorders	6.2 (6.0, 6.4)	2.7 (2.6, 2.7)
Other endocrine disorders	4.0 (3.9, 4.2)	2.5 (2.5, 2.6)
Vitamin and other nutritional deficiencies	3.2 (3.0, 3.3)	3.0 (3.0, 3.1)
Unspecified malnutrition	2.2 (2.1, 2.4)	2.0 (2.0, 2.1)
Hypogammaglobulinemia, agammaglobulinemia, other immunity disorders	1.9 (1.8, 2.0)	1.0 (1.0, 1.1)
Disorders of lipid metabolism	0.9 (0.8, 1.0)	0.6 (0.5, 0.6)
Acquired hypothyroidism	0.8 (0.8, 0.9)	0.7 (0.6, 0.7)
Congenital hypothyroidism	0.6 (0.5, 0.7)	0.4 (0.4, 0.4)

**Table 7:** Rates of the 10 most common conditions in the Hematologic disorders disease category in individuals with asthma age 4 years and younger and the corresponding rates in individuals without asthma.

	Health Services Claims per 1000 person years	
	Individuals with asthma	Individuals without asthma
Condition	(95% confidence interval)	(95% confidence interval)
Iron deficiency anemia	10.5 (10.3, 10.8)	8.8 (8.7, 8.9)
Other diseases of blood, marrow, spleen	4.6 (4.4, 4.8)	3.1 (3.1, 3.2)
Hereditary hemolytic anemia	3.8 (3.7, 4.0)	1.7 (1.7, 1.7)
Purpura, thrombocytopenia, other hemorrhagic conditions	2.2 (2.1, 2.3)	1.6 (1.5, 1.6)
Neutropenia, agranulocytosis, eosinophilia	2.2 (2.0, 2.3)	1.6 (1.6, 1.7)
Coagulation defects	1.9 (1.8, 2.0)	0.8 (0.7, 0.8)
Other anemias	0.9 (0.8, 1.0)	0.9 (0.9, 0.9)
Aplastic anemia	0.6 (0.5, 0.7)	0.5 (0.5, 0.6)
Pernicious anemia	0.3 (0.3, 0.4)	0.3 (0.3, 0.3)
Acquired hemolytic anemia, excluding hemolytic disease of newborn	0.2 (0.2, 0.2)	0.1 (0.1, 0.1)

Among those aged 18 to 44 and 45 to 64 years of age, comorbidity was 47% and 49% higher among those with compared to those without asthma (p<0.001; Figure 2). Among those aged 18 to 44 and 45 to 64 years, physician claims were significantly elevated for all disease categories (p<0.001) except for neoplasms (RR 1.02 [95% CI 0.95 to 1.09] and 0.95 [95% CI: 0.89 to 1.02] respectively, Figure 2). Risk of respiratory disease (other than asthma) was about double, and risk of psychiatric disorders and infectious diseases was approximately 33% to 51% higher in individuals with asthma (p<0.001 for each disease category).

Finally, comorbidity was 22% higher among individuals aged 65 years and older with compared to those without asthma (p<0.001; Figure 2). Although significantly greater health services use was seen in most disease categories, relative risks in this age group were generally lower compared to other age groups. Physician claim rates for respiratory disease (other than asthma) were still higher (RR: 2.08, 95% CI [1.99 to 2.17]) in individuals with asthma, but there was no increase rates for neoplasms, metabolic and immunity disorders, and musculoskeletal disorders and rates for psychiatric disorders was slightly lower (RR: 0.91, 95% CI [0.84 to 0.98]; Figure 2).

### Comorbidity for conditions associated with asthma

Comorbidity, as indicated by physician claims, for several specific conditions previously shown to be associated with asthma was also examined (Tables 4 and 5). Among children four years and younger, physician claim rates for most conditions were significantly higher among those with compared to those without asthma (Figure 3). More than 50% higher claim rates (in some cases more than double) were seen for acute bronchitis, pneumonia, obesity, and behavioral disorders (p<0.001 for each disease condition). Among children aged five to 17 years, physician claim rates for almost all conditions studied was significantly higher among those with compared to those without asthma (Figure 3). More than 50% higher claim rates were seen for acute bronchitis, pneumonia, obesity, and depression (p<0.001 for each disease condition).

Significantly higher physician claim rates for most conditions were found among individuals aged 18 to 44 and 45 to 64 years with asthma compared to those without (p<0.05 for all conditions except lung cancer, osteoporosis and acute myocardial infarction in 45 to 64 year olds; Figure 4). Of particular note were acute bronchitis and pneumonia, which were at least twice as common in those with asthma (p<0.001). Finally, with few exceptions, comorbidity was similar in the 65 years and older age group (Figure 4) where physician claims were twice as common for acute bronchitis and 60% higher for pneumonia.

### Sensitivity Analysis

In the unlikely scenario that individuals who were potentially misclassified as having asthma had twice the comorbidity as those with true asthma, misclassification could have accounted for the results seen. In the more likely scenario that individuals who were potentially misclassified with asthma had less comorbidity than those with true asthma (ie. they were overdiagnosed with asthma), the relative risk of comorbidity would be much greater than that observed [22].

## Discussion

We conducted a retrospective study using universal health administrative data to describe and quantify asthma comorbidity in a large, complete, real world population and found asthma to be associated with significant rates of various types of comorbidity in individuals of different age. Specifically, compared to people without asthma, individuals with asthma had at least 50% or more comorbidity, as indicated by health services use, for respiratory disease (other than asthma) in all age groups; psychiatric disorders in individuals age four and under and age 18 to 44; perinatal disorders in individuals 17 years and under, and metabolic and immunity, and hematologic disorders in children four years and under. While most of these types of asthma comorbidity have been described before,[1], [23]–[26] their differential impact on individuals of different ages adjusting for a number of confounders like socioeconomic status and other comorbidity has, to the best of our knowledge, never been reported. These findings may be used to guide the development of strategies to recognize and manage asthma comorbidity throughout the lifespan to improve asthma care.

Comorbidity may occur in patients with asthma for several reasons and our study was not designed to determine causality. First, asthma itself may cause or contribute to comorbidity. For example, asthma control is often suboptimal and the consequent physical activity limitations and disturbed sleep could contribute to psychiatric disorders and obesity [7], [27], [28]. Second, therapies for asthma may also cause or contribute to comorbidity. For example, inhaled corticosteroids may predispose to pneumonia [29]. Third, some comorbidity such as allergy may be linked to asthma via common genetic and environmental factors as part of a larger diathesis. Fourth, some comorbidity may increase the risk, severity, or likelihood of asthma. This has been well-documented for allergic rhinitis and, more controversially, may also be true for obesity [30], [31]. Finally, it might be possible that some of the comorbidity we observed in individuals with asthma was due to diagnostic confusion or lack of precision.

Most of the disease associations with asthma in the current study have been previously described; however, there were also some, such as metabolic and immunity disorders and hematologic disorders in children 4 years and younger, that we do not believe have been associated with asthma before. This is most likely because they have not been examined before and not because they do not exist. On further examination, differences in the former group appeared to be driven by obesity and diabetes (Table 6) and in the later by iron deficiency anemia (Table 7). Their further investigation would be of interest. There were also a few findings, such as the lack of association with osteoporosis, that appeared to contradict previous studies [1]. This could have been due to differences in measurement of the condition between our study and previous ones and/or differences in analysis–specifically in potential confounding variables adjusted for. In our study we adjusted for many variables including socioeconomic status and other comorbidity which were not adjusted for in previous studies.

The strengths of our study were its ability to examine and quantify all physician health services use in a large real-world, population of individuals of various ages with and without asthma. It also has limitations that merit emphasis. To begin, we used a definition of asthma based on health administrative data, which does not contain a measure of lung function so misclassification could have occurred. This is of greatest concern in the four and under age group because they cannot perform pulmonary function testing making diagnosis of asthma can be very difficult. However, misclassification would have caused the asthma and non-asthma groups we studied to be more similar than they actually were, which would have lessened the differences in comorbidity between the groups and attenuated the relative risks of comorbidity we measured. Thus any misclassification would have caused our study to underestimate (not overestimate) the true impact of comorbidity on individuals with asthma. This was confirmed in our sensitivity analysis which showed that only in the extremely unlikely scenario where those misclassified with asthma had twice the amount of comorbidity as those with true asthma, would the increased relative risks of comorbidity associated with asthma be negated. Our definition of asthma was also likely to overlook milder cases and, because it was not dependent on standardized diagnostic criteria, may have varied in terms of who it identified. A second limitation was that increased health services use may occur for many reasons and our study could not distinguish increased use due to asthma comorbidity from increased use due to other causes such as better access to health care or learned behavior. Even though Ontario has universal health care insurance and we controlled for barriers to access like low socioeconomic status, other obstacles to health care access still exist and individuals who have obtained prior health care access because of their asthma might have been more likely to have found a way to overcome these obstacles. Alternatively, using health care may partly be a learned behavior and those who had accessed the health care system for their asthma might have learned to use, and perhaps overuse, health services for other conditions as well. We believe that these factors may have accounted for a small amount of increased health services use across all categories, but not for the notably higher levels seen in some disease categories. Further study investigating the reasons for increased health services use in individuals with asthma would help to determine if they were contributing factors. Finally, a third limitation was that physicians in Ontario can only provide one diagnosis per claim. Therefore, for example, if a patient presented with asthma and allergic rhinitis (which commonly co-exist) only one would be recorded. This might have accounted for a possible underestimate of the relative risk of allergy in people with asthma.

In summary, we conducted a retrospective cohort study of a large, complete, real world population and found that, compared to people without asthma, those with asthma had notably higher rates of many different types of comorbidity which varied according to age. Asthma comorbidity has important implications in terms of the evaluation and management of asthma and should be recognized, investigated and treated appropriately. Future investigation is needed to confirm and study the unique findings of this study in more detail, sort out the complex interactions between asthma and its comorbidity, and develop better approaches to educating about and managing asthma comorbidity in order to improve asthma care and the health of the asthma population overall.

## Supporting Information

Table S1 **List of disease categories and conditions, their associated International Classification of Disease, 9^th^ Revision (ICD-9) codes, and the specific populations in which they were studied.**
(DOCX)
