# Correction: Describing and Quantifying Asthma Comorbidity: A Population Study

**DOI:** 10.1371/annotation/8743031f-69a0-467b-8231-e74712f4c275

**Published:** 2013-01-31

**Authors:** Andrea S. Gershon, Jun Guan, Chengning Wang, J. Charles Victor, Teresa To

The word "Comorbidity" is misspelled in the article title. The correct title is: Describing and Quantifying Asthma Comorbidity: A Population Study.

The correct citation is: Gershon AS, Guan J, Wang C, Victor JC, To T (2012) Describing and Quantifying Asthma Comorbidity: A Population Study. PLoS ONE 7(5): e34967. doi:10.1371/journal.pone.0034967 

