# Supplementary material for: Describing and Quantifying Asthma Comorbidty: A Population Study
Source: PLoS One. 2012 May 7;7(5):e34967. doi: 10.1371/journal.pone.0034967 (PMC3346768; doi:10.1371/journal.pone.0034967)
Supplement: Table S1 — List of disease categories and conditions, their associated International Classification of Disease, 9th Revision (ICD-9) codes, and the specific populations in which they were studied. (DOCX) [file pone.0034967.s001.docx]

SUPPLEMENTARY MATERIALS

**Supplemental Table 1**. List of disease categories and conditions, their associated International Classification of Disease, 9^th^ Revision (ICD-9) codes, and the specific populations in which they were studied

| **Disease Categories and Conditions** | **International Classification of Disease, 9^th^ Revision (ICD-9) Codes** | **Population studied** |
| --- | --- | --- |
| Disease Categories | | |
| Infectious and parasitic disease | 001.0 – 139.8 | All ages |
| Neoplasms | 140.0 – 239.9 | All ages |
| Endocrine, nutritional, metabolic, and immunity disorders | 240.0 – 279.9 | All ages |
| Hematologic disorders | 280.0 – 289.9 | All ages |
| Psychiatric disorders | 290.0 – 319 | All ages |
| Nervous system and sense organs | 320.0 – 389.9 | All ages |
| Circulatory system | 390.0 – 459.9 | All ages |
| Digestive system | 520.0 – 579.9 | All ages |
| Genitourinary system | 580.0 – 629.9 | All ages |
| Pregnancy, childbirth and the puerperium | 630 – 677 | Females age 18 to 64 years |
| Skin and subcutaneous tissue | 680.0 – 709.9 | All ages |
| Musculoskeletal system and connective tissue | 710.0 – 739.9 | All ages |
| Congenital Anomalies | 740 - 759 | 4 years and younger |
| Conditions originating in the Perinatal Period | 760 - 779 | 4 years and younger |
| Injury and poisoning | 800.0 – 999.9 | All ages |
| Respiratory system other than asthma | 460 – 519 (except 493) | All ages |
| Conditions | | |
| Acute bronchitis | 466 | All ages |
| Pneumonia | 486 | All ages |
| Obesity | 278 | All ages |
| Depression | 311 | All ages |
| Anxiety disorder | 300 | All ages |
| Behavioural disorders | 313 | 17 years and younger |
| Upper respiratory tract infection | 460 | All ages |
| Influenza | 487 | All ages |
| Allergy | 691 – 693 | All ages |
| Angina | 413 | 18 years and older |
| Glaucoma | 365 | 18 years and older |
| Cataracts | 366, E140 (procedure code) | 18 years and older |
| Myocardial infarction | 410, 412 | 18 years and older |
| Osteoporosis | 733 | All |
| Lung cancer | 162 | 18 years and older |

asg
